# Vaccination approach in patients with an allergic reaction to COVID-19 vaccines or at risk of developing allergic reactions

**DOI:** 10.5578/tt.20239920

**Published:** 2023-06-13

**Authors:** Ş. Özden, F.M. Tepetam, Ö. Atik

**Affiliations:** 1 Clinic of Immunology and Allergy, University of Health Sciences Süreyyapaşa Chest Diseases and Thoracic Surgery Training and Research Hospital, İstanbul, Türkiye

**Keywords:** Coronavirus, pandemics, polyethylene glycol

## Abstract

**ABSTRACT:**

Vaccination approach in patients with an allergic reaction to COVID-19 vaccines or at risk of developing allergic reactions

**Introduction:**

There is consensus that patients at risk of developing an allergic reaction to COVID-19 vaccines should be evaluated by an immunologist-allergist to determine whether vaccination should be recommended. We wanted to share our experiences in the management of these high-risk patients, from diagnostic tests in allergological evaluation to the vaccination process.

**Materials and Methods:**

Our retrospective cross-sectional study included patients who had previously developed an allergic reaction to COVID-19 vaccines or drugs and therefore were referred to our allergy and immunology clinic. Prick and intradermal tests were performed on all patients with methylprednisolone acetate (Depo-Medrol®, Pfizer) 40 mg/mL containing polyethylene Glycol (PEG) and triamcinolone acetonide (Kenacort®, Deva) 40 mg/mL containing polysorbate 80. While vaccination with desensitization was recommended for all patients with positive skin tests, split-dose vaccination was recommended for patients with negative skin tests. After explaining the risks and benefits, the choice of the vaccine (Pfizer/BioNTech or Sinovac/ CoronoVac) was left to the patients’ discretion.

**Results:**

A total of 41 patients, 10 males, and 31 females, with a mean age of 42.37 ± 14.177 years were included. Eighteen patients with a history of allergy after COVID-19 vaccines were analyzed according to the type of reaction and type of vaccine administered (Pfizer/BioNTech/Coronovac; Anaphylaxis: 4/1, Urticaria: 11/2). Moreover, there was a history of drug allergy in 23 patients who had not been vaccinated before. Skin tests with PEG were positive in a total of seven patients while skin tests with polysorbate 80 were negative in all patients. No allergic reaction developed in seven patients who underwent desensitization and in 34 patients who received a split dose.

**Conclusion:**

Considering the potentially life-saving benefits of vaccination in a global pandemic environment, it is a safe and effective method to administer vaccines to at-risk patients using desensitization or split dosing techniques, based on their sensitivity status determined through a PEG skin test. This approach allows for the avoidance of preventing access to vaccines, while still ensuring the safety of patients.

## Abstract (tr)

**ÖZ:**

Ağır eozinofilik astımlı hastalarda mepolizumab etkinliği: Bir gerçek yaşam çalışması

**Giriş:**

COVID-19 aşılarına karşı alerjik reaksiyon geliştirme riski olan hastalara aşılamanın önerilip önerilmeyeceğini belirlemek için alerji ve immünoloji uzmanı tarafından bu hastaların değerlendirilmesi gerektiği konusunda fikir birliği vardır. Alerjik değerlendirmede tanısal testlerden aşılama sürecine kadar bu yüksek riskli hastaların yönetimindeki deneyimlerimizi paylaşmak istedik.

**Materyal ve Metod:**

Retrospektif kesitsel çalışmamıza, daha önce COVID-19 aşılarına veya ilaçlara karşı alerjik reaksiyon gelişen bu nedenle alerji ve immünoloji kliniğimize sevk edilen hastalar dahil edildi. Tüm hastalara polietilen glikol (PEG) içeren metilprednizolon asetat (Depo-Medrol®, Pfizer) 40 mg/mL ve polisorbat 80 içeren triamsinolon asetonid (Kenacort®, Deva) 40 mg/mL ile prick ve intradermal testler yapıldı. Deri testi pozitif saptanan tüm hastalara desensitizasyon ile aşılama önerilirken, negatif saptanan hastalara split doz aşılama önerildi. Fayda-risk durumu açıklandıktan sonra hangi aşının seçileceği (Pfizer/BioNTech veya Sinovac/CoronoVac) hastaların kendi tercihine bırakıldı.

**Bulgular:**

Yaş ortalaması 42,37 ± 14,177 olan toplam 41 hasta (10 erkek ve 31 kadın) çalışmaya dahil edildi. COVID-19 aşılarından sonra alerji öyküsü olan 18 hasta reaksiyon tipine ve uygulanan aşı çeşidine göre analiz edildi (Pfizer/BioNTech/Coronovac; anaflaksi:4/1, ürtiker: 11/2). Daha önce aşılanmamış 23 hastada ilaç alerjisi öyküsü vardı. PEG ile yapılan deri testleri toplam yedi hastada pozitif iken, polisorbat 80 ile yapılan deri testleri tüm hastalarda negatifti. Desensitizasyon uygulanan yedi hastada ve bölünmüş doz uygulanan 34 hastada alerjik reaksiyon gelişmedi.

**Sonuç:**

Küresel bir pandemi ortamında aşılamanın potansiyel hayat kurtarıcı faydası düşünüldüğünde, risk altındaki hastaların aşıya erişimini engellemek yerine, hastaların PEG deri testi sonrası duyarlılık durumuna göre desensitizasyon veya split doz ile aşılanması güvenli bir yöntemdir.

## INTRODUCTION

Vaccination is one of the most effective public health interventions in modern medicine. It has been the cure for humanity in the 2019 coronavirus disease (COVID-19). The COVID-19 pandemic not only affected public health but also caused unprecedented international social and economic disruption. While the development of a COVID-19 vaccine has generated excitement, there have been reports of anaphylactic reactions to mRNA vaccines, which have subsequently raised public concerns and the potential for increased vaccine hesitancy within the population. The first patients to develop postvaccination anaphylaxis were healthcare workers in the United Kingdom (UK) who received the COVID-19 vaccine ( 1 ).

Although immediate life-threatening reactions to vaccines are extremely rare, they were reported to occur in 1.3 cases per million doses ( 2 ). Two cases of anaphylaxis became frightening on the second day of the vaccination campaign with a new vaccine (3 3 ).The incidence of anaphylaxis was reported as 2.5 per 10.000 mRNA COVID-19 vaccines in the first prospective real-world cohort of 60.000 employees vaccinated at a large healthcare system (Mass General Brigham-MGB) ( 4 ).

Studies on the safety of CoronaVac are limited. A clinical study in China included 923 patients who received a total of 1.838 doses and reported two hypersensitivity reactions thought to be related to the vaccine in only one patient (urticaria symptom) ( 5 , 6 ).

The cause of allergic reactions to mRNA COVID-19 vaccines is unknown, but the excipient polyethylene glycol (PEG)-2000 found in mRNA COVID-19 vaccines have recently been of considerable interest with limited supporting evidence. PEG is a hydrophilic polymer incorporated in the form of lipidPEG conjugates in both mRNA COVID-19 vaccines from Pfizer/BioNTech and Moderna to stabilize the lipid nanoparticles carrying the mRNA ( 7 , 8 , 9 ). PEG is currently the only excipient in  Pfizer/BioNTech and Moderna vaccines with recognized allergenic potential. PEGs are found in everyday products such as cosmetics, medications, industrial and food products. PEGylation is a process used to extend halflife and limit the volume of distribution of nucleic acid, peptide, and small molecule therapeutics.

In pharmaceuticals, the number included in the name indicates the average molecular weight (e.g., PEG4000). In the cosmetics industry, this number refers to the average number of ethylene oxide units in each molecule (e.g., PEG40). There is also crossreactivity with PEGs and polysorbates ( 10 , 11 ).

To date, there have been no studies conducted that specifically examine the prevalence of PEG hypersensitivity. The onset of serious hypersensitivity reactions and anaphylaxis to PEG is typically rapid and severe. Symptoms include pruritus, flushing, urticaria, and angioedema. It occurs in severe cases with respiratory symptoms such as hypotension, chest tightness, and shortness of breath. The presence of the lipid PEG2000 in both mRNA vaccines (Pfizer/ BioNTech and Moderna) has led to hypotheses suggesting its involvement in anaphylactic reactions.

In Türkiye, the available COVID-19 vaccines include the Pfizer/BioNTech vaccine, which is an mRNA vaccine. Additionally, the country has authorized the use of CoronaVac, an inactivated virus vaccine, and Turkovac, another inactivated vaccine that was introduced at a later stage. The vaccine CoronaVac, developed by Sinovac Life Sciences (Beijing, China), is an inactivated SARS-CoV-2 vaccine developed from African green monkey kidney cells and contains aluminum hydroxide as an adjuvant. Inactivation of SARS-CoV-2 was achieved with β-propiolactone ( 5 ). Although aluminum hydroxide, which is an adjuvant in CoronoVac, is known to cause contact dermatitis, there is no information that it may cause an early allergic reaction.

Within the scope of this study, we detailed our experience with the vaccination approach in patients who developed early allergic reactions (urticaria, anaphylaxis) after the first dose of COVID-19 vaccines (Pfizer/BioNTech or CoronaVac) and in patients with a history of drug hypersensitivity reactions (DHR) with various drugs (oral, subcutaneous, intramuscular or intravenous form) and therefore hesitated to receive COVID-19 vaccine. We would like to share our experience regarding the management of highrisk patients, from diagnostic tests in allergological evaluation to the vaccination process.

## MATERIALS and METHODS

Patients who applied to our institution for an allergic evaluation between 1 September 2021 to 30 December 2021 who had a history of an allergic reaction after the first dose of the COVID-19 vaccine (Pfizer/ BioNTech or CoronaVac) or who were reluctant to be vaccinated due to a previous history of drug hypersensitivity were evaluated.

Demographic and clinical characteristics of the patients were recorded from the hospital data system. The ethics committee of our hospital approved the study (Approval identification number: 258). The study included a total of 41 patients who had a documented history of allergic reactions, such as urticaria and anaphylaxis, following the administration of the first dose of the COVID-19 vaccine. These patients were also hesitant to receive the COVID-19 vaccine due to their previous hypersensitivity reactions to various forms of drugs, including oral and parenteral medications. All patients underwent skin tests (prick and intradermal) with PEG3350 and polysorbate 80 since PEG cross-reacted with polysorbate 80. While applying skin tests, histamine was used as the positive control, and methylprednisolone sodium succinate (Prednol®) 20 mg/mL without PEG and polysorbate was used as the negative control. Triamcinolone acetonide (Kenacort®) 40 mg/mL and methyl-prednisolone acetate (Depo-Medrol®) 40 mg/mL were given for polysorbate 80 and PEG 3350, respectively ( Table 1 ) ( 12 ). When the tests were concluded, the patients were presented with two options for vaccination: Pfizer/BioNTech (mRNA vaccine) or CoronaVac (inactive vaccine).

The contents of these two vaccines are as follows:

### Pfizer/BioNTech

ALC-0315, ALC-0159, polyethylene glycol 2000, 1-2 disterol-sn-glycerol-3 phosphocholine cholesterol, potassium chloride, potassium dihydrogen phosphate, sodium chloride, sodium hydrogen phosphate, disodium hydrogen phosphate, sucrose, water ( 13 ).

### CoronaVac

Aluminum hydroxide, disodium hydrogen phosphate, sodium dihydrogen phosphate, sodium chloride ( 14 ).

**Figure 1:**
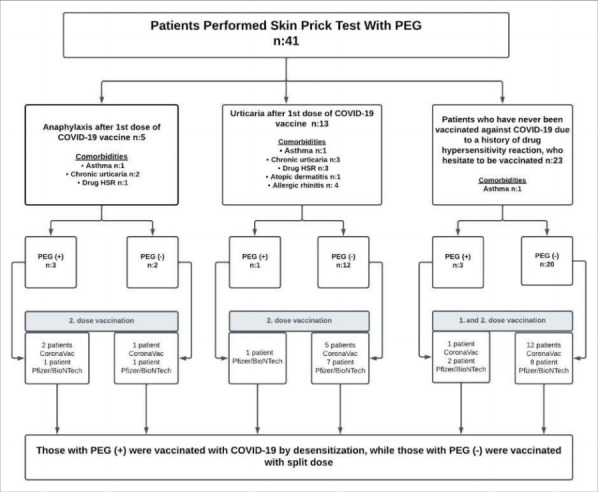
Algorithm that summarizes the procedures included in the study and performed during the vaccination process.

While vaccination with desensitization (with Pfizer/ BioNTech or CoronaVac; Table 2.a and Table 2.b ; respectively) was recommended for all patients with a positive skin test, split dose (1/10, remaining 9/10 if no reaction after 30 minutes) vaccination (with Pfizer/BioNTech or CoronaVac) was recommended for patients with negative results ( 15 , 16 ). The choice of the vaccine was left to the patients. All patients were observed two hours post-vaccination ( Figure 1 ).

### Statistical Analysis

Statistical analyses of the study were performed using the trial version of the SPSS 22.0 (SPSS Inc., Chicago, IL) package software. Descriptive statistics of quantitative variables conforming to normal distribution were shown as mean ± standard deviation. Descriptive statistics for the variables were expressed as frequency (%). A p-value of <0.05 was considered statistically significant.

**Table 1:** Allergometric tests used for patients with suspect polyethylene glycol (PEG) and/or polysorbate 80 (PS80) hypersensitivity

Step		Tested drug	Dilution	Cumulative time (min)
1	Positive control	Histamine	1:1	0
	Negative control	Methyl-prednisolone sodium succinate (Prednol) 20 mg/mL	1:1	
	Prick test	Methyl-prednidolone Acetate (Depo-Medrol) 40 mg /mL	1:100	
	Prick test	Triamcinolone acetonide (Kenacort) 40 mg/mL	1:100	
2	Prick test	Methyl-prednidolone Acetate (Depo-Medrol) 40 mg /mL	1:10	30
	Prick test	Triamcinolone acetonide (Kenacort) 40 mg/mL	1:10	
3	Prick test	Methyl-prednidolone Acetate (Depo-Medrol) 40 mg /mL	1:1	60
4	Intradermal	Methyl-prednidolone Acetate (Depo-Medrol) 40 mg /mL	1:1000	90
	Intradermal	Triamcinolone acetonide (Kenacort) 40 mg/mL	1:1000	
5	Intradermal	Methyl-prednidolone Acetate (Depo-Medrol) 40 mg /mL	1:10	120
	Intradermal	Triamcinolone acetonide (Kenacort) 40 mg/mL	1:10	
6	Observation			180

**Table 2:** Pfizer/BioNTech desensitization protocol

	Dose (mL)	Total Dose (mL)
Step 1	0.03	0.3
Step 2	0.07	0.1
Step 3	0.10	0.2
Step 4	0.10	0.3

30 minutes between steps.

**Table 3:** CoronoVac desensitization protocol

	Dose (mL)	Total Dose (mL)
Step 1	0.05	0.05
Step 2	0.05	0.1
Step 3	0.1	0.2
Step 4	0.15	0.35
Step 5	0.15	0.5

30 minutes between steps.

## RESULTS

A total of 41 patients (10 male and 31 female) were included in the study. The mean ages of males and females were 38.80 ± 12.145 and 43.52 ± 14.769 years, respectively. While 2 (4.8%) patients had no comorbidities, 27 (65.8%) had a history of drug hypersensitivity (oral or parenteral), 5 (12.1%) had chronic urticaria, 4 (9.7%) had allergic rhinitis, 3 (7.3%) had asthma and 1 (2.4%) had atopic dermatitis.

Eighteen patients developed an allergic reaction (anaphylaxis n= 5, urticaria n= 13) after the first dose of the COVID-19 vaccine (Pfizer/BioNTech or CoronaVac), and 23 patients had never been vaccinated against COVID-19 before. When the medical history of 23 patients was examined, all had a history of drug hypersensitivity to various forms (oral, intramuscular, intravenous, subcutaneous, etc.) of various drugs (non-steroidal anti-inflammatory drugs, antibiotics, etc.). Due to their previous medical history, these 23 patients expressed hesitation towards receiving the newly developed COVID-19 vaccination plan.

PEG and Polysorbate 80 skin tests were performed on all 41 patients. Skin tests with polysorbate 80 were negative in all patients. The PEG skin test was positive in three of five patients with a history of anaphylaxis and one of 13 patients with a history of urticaria after the first dose of the COVID-19 vaccine. Of the 23 patients with a history of drug hypersensitivity reaction who had never been vaccinated against COVID-19, three had a positive PEG skin test.

In this study, PEG (+) was detected in three of five patients who developed anaphylaxis after the first dose of the COVID-19 vaccine, and the choice of the vaccine in the second dose was (Pfizer/BioNTech or CoronaVac) presented to the patient’s initiative. While two of three patients wanted to be vaccinated with CoronaVac, one chose the Pfizer/BioNTech vaccine. Among the patients, one individual with a positive PEG history and a clinical history of developing urticaria after receiving the Pfizer/BioNTech COVID-19 vaccine chose to receive Pfizer/BioNTech for the second dose. Of the three patients with a history of drug hypersensitivity reactions (HSR) who were PEG positive, two opted for the Pfizer/BioNTech vaccine for the second dose, while one chose CoronaVac for the second dose. Patients who tested positive on skin tests were administered desensitization protocols, while patients who tested negative were given split-dose vaccinations. No allergic reactions were observed in patients who were monitored for a period of two hours following both the split dose vaccination and the desensitization procedures ( Figure 1 ).

## DISCUSSION

The development of vaccines against COVID-19 has instilled hope that the global pandemic will finally come to an end. Vaccination is the most powerful tool against the pandemic.  However, the occurrence of anaphylaxis requiring epinephrine intervention in two patients on the day of receiving mRNA vaccines has created a bias against vaccines ( 17 ). As a result, patients with a prior history of allergic reactions approached COVID-19 vaccination with skepticism. However, it was crucial to vaccinate as many people as possible in order to effectively combat the pandemic. Convincing individuals about the safety of vaccination proved to be the most challenging task for healthcare workers ( 18 , 19 ). The cause of COVID-19 vaccine allergic reactions is still unclear. Allergic reactions to vaccines are usually caused by adjuvants and preservatives and other excipients/components in the vaccine rather than the active substance itself ( 20 ).

This study discussed 41 patients referred to the allergy clinic for pre-vaccination evaluation who developed various allergic reactions after the first COVID-19 vaccine and/or drugs. All patients received vaccination using desensitization or split-dose protocols, depending on the results of the skin tests and considering the excipients present in the COVID-19 vaccines. No adverse events or complications were observed in any of the vaccinated patients.

While it is not standard practice to perform a skin prick test specifically for polyethylene glycol (PEG) and polysorbate before vaccination in patients with drug hypersensitivity, it is worth noting that these agents are commonly found as excipients in various medications, including tablets, topical gels, parenteral steroids, laxatives, and others. Therefore, the use of a PEG skin test is considered to be useful in guiding the administration of the second dose of vaccination in patients who experience an allergic reaction following the initial dose of mRNA COVID-19 vaccines ( 15 ). Due to the presence of numerous excipients in the COVID-19 vaccines and the impracticality of testing against all of them, a skin test was conducted specifically targeting PEG, which is considered the most allergenic ingredient, and polysorbate, which can potentially cross-react with PEG. It was not feasible to perform testing using the vaccines themselves due to limited access caused by the pandemic conditions.

In our study, PEG (+) was detected in 7 (17.07%) of 41 patients in whom we performed PEG skin testing due to COVID-19 vaccine allergy or drug allergy history, while polysorbate 80 positivity was not detected in any patient. In the study by AlMuhizi et al. ( 16 ) 142 patients with a history of an allergic reaction after the first dose of vaccination with mRNA COVID-19 such as Moderna or Pfizer/BioNTech were examined and PEG skin prick test (+) was found in only one patient, while polysorbate 80 positivity was not detected, similar to our study. However, intradermal testing was not performed in this study. In the study by Wolfson et al. ( 21 ) in which intradermal tests were also used in the evaluation of PEG allergy as in our study, 80 patients with a history of mRNA COVID-19 vaccine reaction were evaluated and PEG (+) (6.25%) was found in five patients and polysorbate 80 (+) (15%) in 12 patients. Polysorbate 80, which can cross-react with PEG, is used as an excipient in AstraZeneca and Johnson & Johnson vaccines, which are not available in our country. Differences in the frequency of polysorbate 80 skin sensitization in the studies may be due to this reason. None of the patients vaccinated with CoronaVac, which does not contain PEG or polysorbate 80, had a positive skin test for these ingredients. However, the positive PEG skin test in three patients with a history of drug HSR who had never been exposed to the COVID-19 vaccine may be explained by previous exposure to different drugs or cosmetics containing PEG.

In our study, it has been shown that COVID-19 vaccines can be administered safely and successfully with desensitization or split dose to patients who are hesitant to be vaccinated due to previous allergic reactions. Among the 142 high-risk patients of AlMuhizi et al. ( 16 ) one of six patients who underwent desensitization for reasons such as PEG allergy and/ or drug allergy and/or atopic disease developed urticaria on 3-4 days of vaccination, but this was attributed to the activation of the patient’s pre-existing chronic urticaria. Maculopapular eruption developed in one patient. Morbilliform rashes, with a reported incidence of up to 7%, have been documented with COVID-19 vaccines ( 22 ). In our study, five of seven patients who underwent desensitization had chronic urticaria, and no allergic reaction developed during desensitization or in the following days. No maculopapular or morbilliform rash was observed in any of our patients. Our desensitization success may depend on our protocol including more steps.

In the study mentioned above conducted by Wolfson et al. ( 21 ) in which 80 patients with a history of COVID-19 vaccine reaction were evaluated, 2nd dose vaccination was administered directly to 70 patients without desensitization. Regardless of PEG or polysorbate 80 sensitivity, 62 (89%) patients reported either no reaction or a mild reaction that could be controlled with antihistamines. However, it was emphasized that epinephrine treatment was required in two patients.

Considering the possibility of a rare but life-threatening reaction, it would be safer to administer split doses in patients with a history of anaphylaxis or severe allergic reactions to vaccines as recommended by the European Academy of Allergy & Clinical Immunology (EAACI) ( 23 ). In our study, it was shown for the first time that this method of administration can also be a reliable option for COVID-19 vaccination.

**Figure 2:**
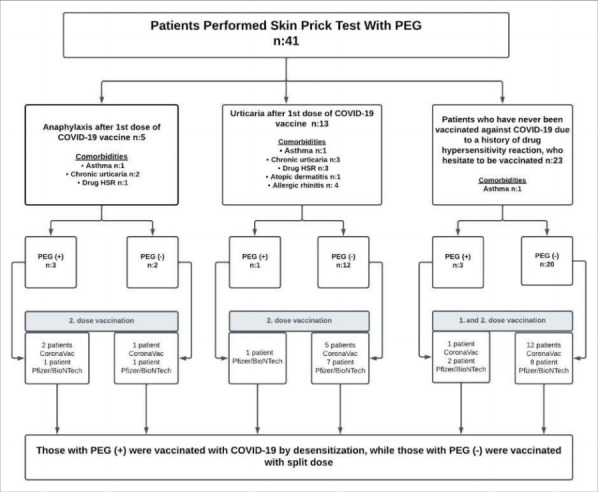
Management of patients who present with symptoms concerning for an allergic reaction to the first dose of mRNA COVID-19 vaccine. Use of Janssen vaccine (if available) may be appropriate after allergic reaction to the first dose of mRNA COVID. *Same COVID-19 vaccine manufactured as first dose. †Limited supply of Janssen COVID vaccine; only use when clinically necessary.

We implemented our algorithm for managing patients who developed allergic reactions after the first dose of mRNA COVID-19 vaccination, drawing inspiration from the approach established by Wolfson et al. ( 21) (Figure 2). This algorithm emphasizes the importance of the patient’s clinical history and the evaluation conducted by the allergist as key steps in the management process. After risk-stratifying the patients based on their reaction history, it was determined that the majority of low and medium-risk patients could proceed with the second dose without encountering

However, vaccine hesitancy is a major problem in patients with a history of mild reactions or drug allergies, even in the absence of anaphylaxis. COVID-19 can cause serious symptoms, and vaccination against COVID-19 is an important global challenge, many people need to be vaccinated safely. Patients almost want assurances from health professionals about the safety of the vaccine.

**Figure 3:**
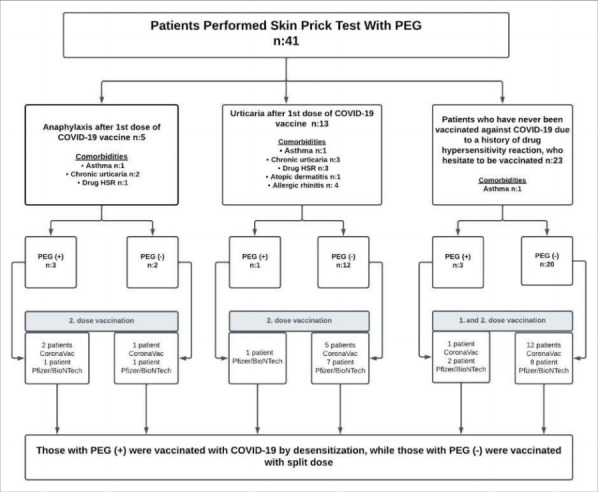
Approach algorithm for COVID-19 vaccine reactions that we apply in clinical practice.

In this study, we aimed to share our clinical experience regarding our approach to patients who have an allergic reaction after the first dose of the COVID-19 vaccine or who have a history of various allergic reactions to drugs, which we encounter quite frequently in daily practice and, therefore seriously refrain from being vaccinated. We have created a practical algorithm to guide physicians in the management of these high-risk patients referred to immunology and allergy specialists. The approach including desensitization/split dose preference in vaccination according to the results of diagnostic skin tests with PEG and polysorbate 80 and the clinical risk level of the patients is summarized in Figure 3.

## Ethical Committee Approval

This study was approved by University of Health Sciences Süreyyapaşa Chest Diseases and Thoracic Surgery Training and Research Hospital Ethics Committee (Decision no: 258, Dacision date: 20.01.2022).

## CONFLICT of INTEREST

The authors declare that they have no conflict of interest.

## AUTHORSHIP CONTRIBUTIONS

Concept/Design: All of authors

Analysis/Interpretation: All of authors

Data acqusition: All of authors

Writing: All of authors

Clinical Revision: All of authors

Final Approval: All of authors
